# Anticipatory attention is a stable state induced by transient control mechanisms

**DOI:** 10.3389/fnhum.2022.965689

**Published:** 2022-07-22

**Authors:** Sean Noah, Sreenivasan Meyyappan, Mingzhou Ding, George R. Mangun

**Affiliations:** ^1^Center for Mind and Brain, University of California, Davis, Davis, CA, United States; ^2^Helen Wills Neuroscience Institute, University of California, Berkeley, Berkeley, CA, United States; ^3^J. Crayton Pruitt Family Department of Biomedical Engineering, University of Florida, Gainesville, FL, United States; ^4^Department of Psychology, University of California, Davis, Davis, CA, United States; ^5^Department of Neurology, University of California, Davis, Davis, CA, United States

**Keywords:** attention, EEG, decoding, alpha, ERP, cue, object, SVM—support vector machine

## Abstract

Anticipatory attention is a neurocognitive state in which attention control regions bias neural activity in sensory cortical areas to facilitate the selective processing of incoming targets. Previous electroencephalographic (EEG) studies have identified event-related potential (ERP) signatures of anticipatory attention, and implicated alpha band (8–12 Hz) EEG oscillatory activity in the selective control of neural excitability in visual cortex. However, the degree to which ERP and alpha band measures reflect related or distinct underlying neural processes remains to be further understood. To investigate this question, we analyzed EEG data from 20 human participants performing a cued object-based attention task. We used support vector machine (SVM) decoding analysis to compare the attentional time courses of ERP signals and alpha band power. We found that ERP signals encoding attentional instructions are dynamic and precede stable attention-related changes in alpha power, suggesting that ERP and alpha power reflect distinct neural processes. We proposed that the ERP patterns reflect transient attentional orienting signals originating in higher order control areas, whereas the patterns of synchronized oscillatory neural activity in the alpha band reflect a sustained attentional state. These findings support the hypothesis that anticipatory attention involves transient top-down control signals that establish more stable neural states in visual cortex, enabling selective sensory processing.

## Hypothesis

With anticipatory attention, the brain prioritizes incoming stimuli relevant to behavioral goals. To accomplish this, attention control regions in the brain increase excitability of sensory cortical areas responsive to targeted stimuli. It is unknown whether attention control regions influence sensory regions with sustained input, or if instead a transient control signal is sufficient to induce a stable, sensitive sensory state. We collected electroencephalography data while participants performed anticipatory attention. Using machine learning classification, we found that two signals of interest—the event-related potential (ERP) signal and EEG alpha band power—reflect distinct phases of an anticipatory attention process, with different stability properties. We hypothesize that ERP reflects a transient control signal and alpha power reflects a stable, modulated sensory state.

## Introduction

Attention is the selective prioritization of sensory or cognitive information that is relevant to behavioral goals. Endogenous attention, in which “top-down” control is a key component, is the volitional focusing of processing capacity on goal-relevant sensory or cognitive targets (Corbetta and Shulman, 2002). The effort involved in deliberately directing processing resources distinguishes endogenous attention from exogenous (bottom-up) attention that is driven by sensory properties such as salience (Itti, 2005; Geng and Mangun, 2009) and meaning (Henderson and Hayes, 2017), and other forms of attention driven by selection history and reward association (Awh et al., 2012; Anderson, 2016; Failing and Theeuwes, 2018).

Endogenous attention can be deployed in anticipation of an upcoming sensory stimulus (Posner, 1980). In real life, for example, when someone in a hurry waits for a traffic light to turn green, they are effortfully monitoring the traffic light to the exclusion of other potentially salient stimuli in their environment so that as soon as the light changes, they can speed onward. They do not know when the light will change, so their anticipation of the green light induces a steady state of heightened and selective attentiveness, readying their visual system to detect the target stimulus and coordinate the appropriate motor response with minimal delay.

Research conducted with electroencephalography (EEG) methods has revealed multiple scalp-level signatures of anticipatory attention. Examining phase-locked voltage fluctuations in trial-averaged EEG data relative to the onset of an experimental event such as the attention cue has uncovered differences among the condition-averaged ERP waveforms attributable to different orienting processes (Dale et al., 2008), including an early contralateral positivity (Simpson et al., 2006), an early attention-directing negativity (Harter et al., 1989; Nobre et al., 2000), an anterior attention-directing negativity (Simpson et al., 2006), and a late directing attention positivity (Hopf and Mangun, 2000). Functional MRI and seeded source modeling have been used to associate different ERP signatures with brain areas that constitute an attention control network (Grent-‘T-Jong and Woldorff, 2007).

Alpha band (8—12 Hz) power modulation, inversely indexing the change of cortical excitability, has also been associated with anticipatory attention (Jensen and Mazaheri, 2010; Van Diepen et al., 2019). In covert visual spatial attention, alpha band power has been found to be relatively lower over the visual cortex contralateral to the attended hemifield (Worden et al., 2000; Sauseng et al., 2005). In feature-based attention, alpha power modulation has also been observed in feature processing areas (Snyder and Foxe, 2010).

In our previous work, we showed that multivariate patterns of alpha power distributions carry information about the object category that is in the current focus of attention (Noah et al., 2020). In that study, we implemented a cued, object-based attention paradigm in which, on each trial, participants anticipated an upcoming target object image after being cued to the expected object category, and were instructed to perform a perceptual judgment about the target object image when it appeared. Using a support vector machine (SVM) decoding approach, we observed that EEG alpha band (8–12 Hz) power topographies were systematically modulated in the late-cue period, corresponding to the object category being anticipated. We interpreted this finding to support a model of visual attention in which modulation of alpha band oscillatory neural activity plays a crucial role in facilitating processing of anticipated, task-relevant visual information. In that study, we did not investigate the relationship between ERP signatures of anticipatory object-based attention and alpha band modulation, and so here we address that topic.

The extent to which anticipatory attention-related ERP components and alpha band modulation are related to the same or different underlying neural mechanisms of attention remains poorly understood. ERPs phase-locked to an experimental event and ongoing neural oscillations are distinct neural activities with different generating mechanisms (Shah et al., 2004). As such, it seems likely that occipital scalp-recoded ERPs and alpha power patterns related to attentional control index different neural mechanism of attentional control. Indeed, attention-related ERP components have been localized to frontal brain areas (Grent-‘T-Jong and Woldorff, 2007) and occipital areas (Hopf and Mangun, 2000; Hong et al., 2015), whereas alpha power asymmetries with spatial attention are consistent with neural generators in occipital cortex (Worden et al., 2000; Snyder and Foxe, 2010).

These arguments notwithstanding, the possible overlap between some ERP measures of anticipatory attention and alpha band modulation cannot be entirely ruled out. For example, sustained voltage deflections over visual cortex contralateral to the covertly attended hemifield could index a similar mechanism as alpha band modulation: decreased alpha band power is thought to correspond to increased baseline excitability (Jensen and Mazaheri, 2010), and this excitability could also be reflected in the phase-locked EEG signal as a sustained voltage increase. Thus, empirical studies are required to examine the extent of overlap between ERP measures of anticipatory attention and alpha band measures.

To investigate the relationship between ERP and EEG alpha measures of attentional control and visual cortical biasing during anticipatory attention, we analyzed EEG from human participants who had performed an anticipatory object-based attention task. We applied SVM classification to compare the time courses of ERP decoding and alpha decoding during selective visual attention.

## Methods

The design of this experimental paradigm is identical to that of previously published work (Noah et al., 2020). In the study presented here, we report data from a larger number of participants and utilize different analysis methods to probe our hypothesis about the relationship between ERP measures of attentional control and oscillatory signals related to visual cortical biasing.

### Participants

All participants were healthy undergraduate and graduate student volunteers from University of California, Davis, had normal or corrected-to-normal vision, gave informed consent in accordance with the University of California, Davis, Institutional Review Board, and received monetary compensation for their time.

EEG data were recorded from 23 healthy undergraduate and graduate student volunteers (11 males and 12 females). EEG datasets from three participants were excluded because of irreconcilable noise in the EEG data or subject non-compliance with the task requirements, yielding a final EEG dataset including 20 participants (9 males and 11 females). This sample size was chosen based on prior experiments that found significant above chance decoding in the alpha band EEG signal during an anticipatory object-based attention task (Noah et al., 2020).

### Apparatus and stimuli

Participants were comfortably seated in an electrically shielded, sound-attenuating room (ETS-Lindgren, United States). Stimuli were presented on a VIEWPixx/EEG LED monitor, model VPX-VPX-2006A (VPixx Technologies Inc., Quebec Canada), at a viewing distance of 85 cm, vertically centered at eye level. The display measured 23.6 inches diagonally, with a native resolution of 1,920 by 1,080 pixels and a refresh rate of 120 Hz. The recording room and objects in the room were painted black to reduce reflected light. The recording room was dimly illuminated using DC lights.

**FIGURE 1:**
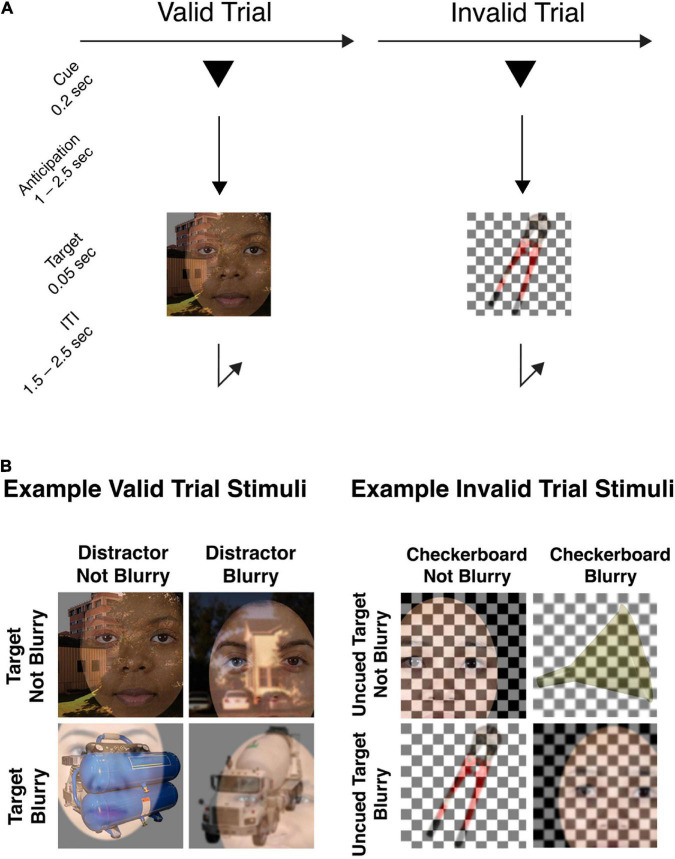
**(A)** Example trial sequence. Each trial began with the presentation of a symbolic cue that was predictive of the upcoming object category (80%). Following an anticipation period (cue-to-target) varying from 1.0 to 2.5 s, a composite stimulus image was presented. Participants were required to make a rapid-accurate discrimination of whether the cued object image was blurry or not-blurry (valid trials), or whether the uncued object image was blurry or not-blurry (invalid trials). **(B)** Example stimulus images in the attention task. In the set of example valid trial stimuli shown, Face is the target object category to be identified as in-focus or blurry, and the overlaid Tool or Scene images are the distractor images. For each stimulus image, both the target and distractor can be blurry or in-focus, independently of each other. Example invalid trial stimuli are also provided to illustrate that both the uncued target image and the overlaid checkerboard can be blurry or not-blurry, independently of one another. In the invalid trial condition, participants were still trained to respond to the uncued target image with the same blurry/not-blurry distinction, using the same response buttons as for valid trials.

The behavioral task for this experiment was to determine, on each trial, whether the briefly presented target image belonging to the cued object category (face, scene, or tool) was in-focus or blurry. The trial sequence is presented in Figure 1A.

The stimuli to be discriminated were composites of an image belonging to the target category superimposed with an image belonging to a non-cued, distractor category. Crucially, both the target image and the distractor image in the composite image could be in-focus or blurry independently of each other (Figure 1B), therefore, the task could not be performed solely by attending to and responding to the presence or absence of blur. As well, the stimulus parameters were such that task difficulty was high, and successful discrimination of focus/blur therefore required anticipatory attentional effort.

Each trial began with the pseudorandomly selected presentation of one of three possible cue types for 200 ms (1°× 1° triangle, square, or circle, using PsychToolbox; Brainard, 1997). Valid cues informed participants which target object category (face, scene, or tool, respectively) was likely to subsequently appear (80% probability) and instructed participants to attend to the object image from that category. Cues were presented 1° above the central fixation point. Following pseudorandomly selected SOAs (1,000–2,500 ms) from cue onset, target stimuli (5°× 5° square image) were presented at fixation for 50 ms. We used variable SOAs to maximize the incentive for participants to engage sustained anticipatory attention during the cue-target interval. For a discussion of tradeoffs involved in experimental designs with variable cue-target intervals, see (Vallesi, 2010).

Twenty percent of trials were invalidly cued, allowing us to assess the effect of cue validity on behavioral performance. For the invalid trials, the stimulus image was a composite of an image from a randomly chosen non-cued object category, superimposed with a black and white checkerboard. The checkerboard could also be blurry or in-focus independently of the object image. Participants were instructed that whenever they encountered a trial where the blended stimulus didn’t include an image belonging to the cued object category, but instead contained only one object image and a checkerboard overlay, then they had to indicate whether the non-cued object image in the stimulus was blurry or in-focus. We predicted that participants would be slower to respond on invalidly cued trials, analogously to the behavioral effect of validity observed in cued spatial attention paradigms.

The stimulus images spanned a square 5°× 5° of visual angle. To create blurred images, Gaussian blur with a standard deviation of 2 was applied to the images. All stimuli were presented against a gray background. A white fixation dot was continuously present in the center of the display.

All three object categories included 40 different individual images. On each trial, random images were drawn to produce the composite stimulus image. All target images were gathered from the Internet. Face images were front-facing and neutral-expression, cropped and placed against a white background (Ma et al., 2015). All face images were cropped to ovals centered on the face and placed against a white background. Full-frame scene images were drawn from the University of Texas at Austin’s natural scene collection (Geisler and Perry, 2011) and campus scene collection (Burge and Geisler, 2011). Tool images, cropped, and placed against a white background, were drawn from the Bank of Standardized Stimuli (Brodeur et al., 2014). Unlike scene images, which contained visual details spanning the entire 5°× 5° square, face and tool images were set against white backgrounds and so did not contain visual information up to all the image boundaries. Therefore, to eliminate the possibility that participants could use cue information to focus spatial attention instead of object-based attention to perform the blurry/in-focus discrimination, on any trial where a face or tool image was included in the composite stimulus, the position of that face or tool image was randomly jittered from the center.

A pseudorandomly distributed inter-trial-interval (ITI; 1,500–2,500 ms) separated target offset from the cue onset of the next trial.

### Procedure

Participants were instructed to maintain fixation on the center of the screen during each trial, and to anticipate the cued object category until the target image appeared. They were further instructed to indicate whether the target image was blurry or not-blurry with a button press as quickly as possible upon target presentation, using the index finger button for “blurry” and the middle finger button for “not-blurry.” Responses were only recorded during the ITI between target onset and the next trial. Trials were classified as correct when the recorded response matched the target image subcategory, and incorrect when the response did not match, or when there was no recorded response.

Participants were instructed to respond as quickly as they could to the target stimulus, making it vital that the participants engaged preparatory attention toward the cued object category during the preparatory period. All participants were trained with at least 42 trials of the task and were able to achieve at least 60% response accuracy before performing it under EEG data collection; to achieve this, stimulus duration was adjusted on an individual participant basis during the initial training phase to facilitate training on the task.

Each participant completed 15 blocks of the experiment, with each block comprising 42 trials, totaling 630 trials.

### Electroencephalography recording

Raw EEG data were acquired with a 64-channel Brain Products actiCAP active electrode system (Brain Products GmbH) and digitized using a Neuroscan SynAmps2 input board and amplifier (Compumedics USA, Inc.). Signals were recorded with Scan 4.5 acquisition software (Compumedics USA, Inc.) at a sampling rate of 1,000 Hz and a DC to 200 Hz online band pass. Sixty-four Ag/AgCl active electrodes were placed in fitted elastic caps using the following montage, in accordance with the international 10-10 system (Jurcak et al., 2007): FP1, FP2, AF7, AF3, AFz, AF4, AF8, F7, F5, F3, F1, Fz, F2, F4, F6, F8, FT9, FT7, FC5, FC3, FC1, FCz, FC2, FC4, FC6, FT8, FT10, T7, C5, C3, C1, Cz, C2, C4, C6, T8, TP9, TP7, CP5, CP3, CP1, CPz, CP2, CP4, CP6, TP8, TP10, P7, P5, P3, P1, Pz, P2, P4, P6, P8, PO7, PO3, POz, PO4, PO8, PO9, O1, Oz, O2, PO10; with channels AFz and FCz assigned as ground and online reference, respectively. Additionally, electrodes at sites TP9 and TP10 were placed directly on the left and right mastoids. The Cz electrode was oriented to the vertex of each participant’s head by measuring anterior to posterior from nasion to inion, and right to left between preauricular points. High viscosity electrolyte gel was administered at each electrode site to facilitate conduction between electrode and scalp, and impedance values were kept below 25 kΩ. Continuous data were saved in individual files corresponding to each trial block of the stimulus paradigm.

### Electroencephalography preprocessing

All data preprocessing procedures were completed with the EEGLAB Matlab toolbox (Delorme and Makeig, 2004). For each participant, all EEG data files were merged into a single dataset before data processing. Each dataset was visually inspected for the presence of bad channels, and any bad channels identified were subject to data interpolation from neighboring electrodes. The data were Hamming window sinc FIR filtered (1–83 Hz), and then down sampled to 250 Hz. Data were algebraically re-referenced to the average of all electrodes, and then further low pass filtered to 40 Hz. Data were epoched from 500 ms before cue onset to 1,000 ms after cue onset, so that anticipatory data from all trials could be examined together. Data were visually inspected to flag and reject trials with muscle tension artifact and eye movement artifacts that occurred during cue presentation. Independent component analysis (ICA) decomposition was then used to remove artifacts associated with blinks and eye movements.

### Electroencephalography decoding analysis

We implemented a decoding analysis to quantitatively assess whether object-based attention was systematically associated with changes in phase-independent alpha band (8–12 Hz) power topography and phase-dependent ERP across conditions. The data used for the ERP decoding was restricted to signals below 6 Hz in frequency, to minimize the overlap in information with the alpha band data. This analysis routine was adapted from a routine to decode working memory and attention representations from scalp EEG (Bae and Luck, 2018; Noah et al., 2020). To isolate the 8–12 Hz alpha band signal from the EEG, we used a power spectral density procedure, with the Matlab *periodogram()* function (window length 500 ms, step length 40 ms). Bandpass filtered alpha band signal was subjected to Hilbert transform to approximate instantaneous power in this band. Thus, we proceeded to decode two separate signals over 64 electrodes: 0–6 Hz ERP and 8–12 Hz alpha band power.

Decoding was performed independently at each time point within the epochs. We implemented our decoding model with the Matlab *fitcecoc()* function to use the combination of a SVM and error-correcting output coding (ECOC) algorithms. A separate binary classifier was trained for each cue condition (attend-face, attend-scene, or attend-tool), using a one-vs.-one approach, with classifier performance combined by the ECOC algorithm. Thus, decoding was considered correct when the classifier correctly determined the cue condition from among the three possible cue conditions, and chance performance was set at 33.33% (one-third).

The decoding for each time point followed a sixfold cross-validation procedure. Data from five-sixths of the trials, randomly selected, were used to train the classifier with the correct labeling. The remaining one-sixth of the trials were used to test the classifier, using the Matlab *predict()* function. This entire training and testing procedure was iterated 10 times, with new training and testing data assigned randomly in each iteration. For each cue condition, each participant, and each time point, decoding accuracy was calculated by summing the number of correct labelings across trials and iterations and dividing by the total number of labelings.

We averaged together the decoding results for all 10 iterations to examine decoding accuracy across participants, at every time point in the epoch. At any given time point, above-chance decoding accuracy suggests that the decoded signal contains information about the attended object category. We utilized a Monte Carlo simulation-based significance assessment to reveal statistically significant clusters of decoding accuracies. Full details of this cluster-based permutation test for statistical significance are described in previous publications describing how this method was applied in other experiments (Bae and Luck, 2018; Noah et al., 2020).

We conducted a cross-temporal decoding analysis to measure the extent to which decoded patterns of EEG voltages and oscillatory power were stable over time. Our cross-temporal decoding procedure involved classifying each test data time point for each train data time point. For each time point, training set data was used to generate a classifier model, and then data from each time point in the testing set was subjected to classification by the trained model. Thus, the ability of a classifier trained at one time point to perform above-chance decoding on test data from other time points indicates that the topographic pattern of voltages or oscillatory power over which the model was trained was equivalent across the time points in question.

## Results

**FIGURE 2:**
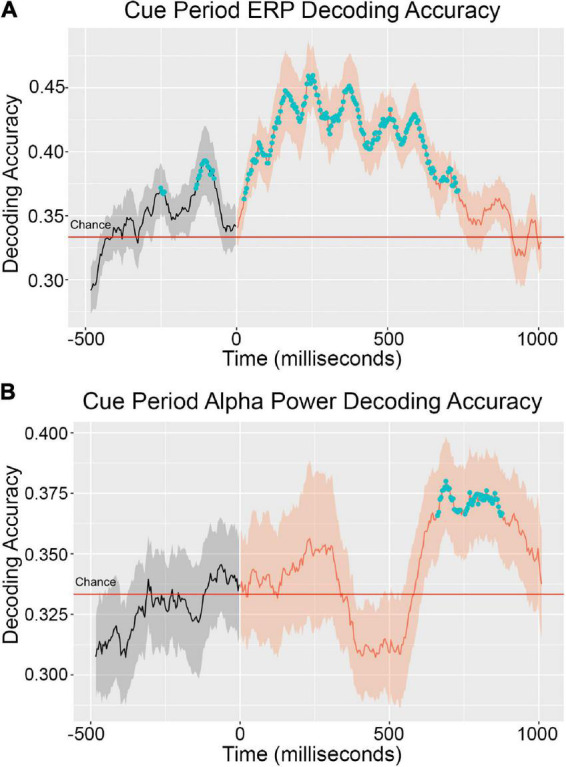
Cue period decoding accuracy timeseries for ERP **(A)** and alpha power **(B)**. Decoding accuracy was averaged across EEG data from 20 participants. The solid line inside the shaded regions represents mean decoding accuracy across participants and the shading represents the standard error across participants. The blue dots denote clusters of time points statistically significantly greater than chance (33%). The ERP signal was extracted from the original EEG signal by low pass filter with a 6 Hz cutoff. Instantaneous alpha power was calculated with a Hilbert transform over 8–12 Hz bandpass filtered EEG data.

The ERP decoding timeseries is presented in Figure 2A. In the cue period between 0 and 1,000 ms after cue onset, a cluster of statistically above-chance time points extended from 0 to 750 ms. Average decoding accuracy across participants peaked around 200 ms at about 45% (33% is the chance level). Decoding accuracy remained at roughly the same level to 400 ms, and then began to steadily decline. Above-chance level decoding prior to stimulus onset (*t* = 0) is likely an artifact due to narrow-band filtering (0–6 Hz). The reason for choosing 6 Hz as the upper limit of the passing band was to avoid the influence of alpha oscillations (8–12 Hz) on the ERP analyses. According to the theory of spectral filtering, the narrower the filtering band, the more severe the temporal smearing.

It is common in the EEG decoding literature for absolute decoding accuracy values to be close to chance. Therefore, statistically significant difference from chance is taken to be the primary indicator of information about the decoded conditions being present in the decoded signal (Bae and Luck, 2018; Noah et al., 2020). That absolute decoding accuracies cannot easily be interpreted as continuous measures of condition information in decoded signals is a limitation of the EEG decoding method.

The alpha power decoding timeseries is presented in Figure 2B. In the cue period, average decoding accuracy across participants did not reach the level of statistically significant above-chance performance until 625 ms post cue onset. Statistically significant above-chance decoding occurred from this time until 875 ms. Decoding performance began dropping off after this point but remained above chance levels. In the early phase of the cue period, decoding performance trended above-chance in the window of 0–300 ms but did not reach the level of statistical significance. It is possible that above-chance decoding performance in this early window is attributable to the different sensory responses to the three different cue shapes that accompanied each object attention condition. In a previous study, we examined the extent to which above-chance alpha power decoding could be achieved solely based on physical differences in the cue shape and localized shape-driven above-chance performance to the early window of 0–300 ms post cue onset (Noah et al., 2020).

**FIGURE 3:**
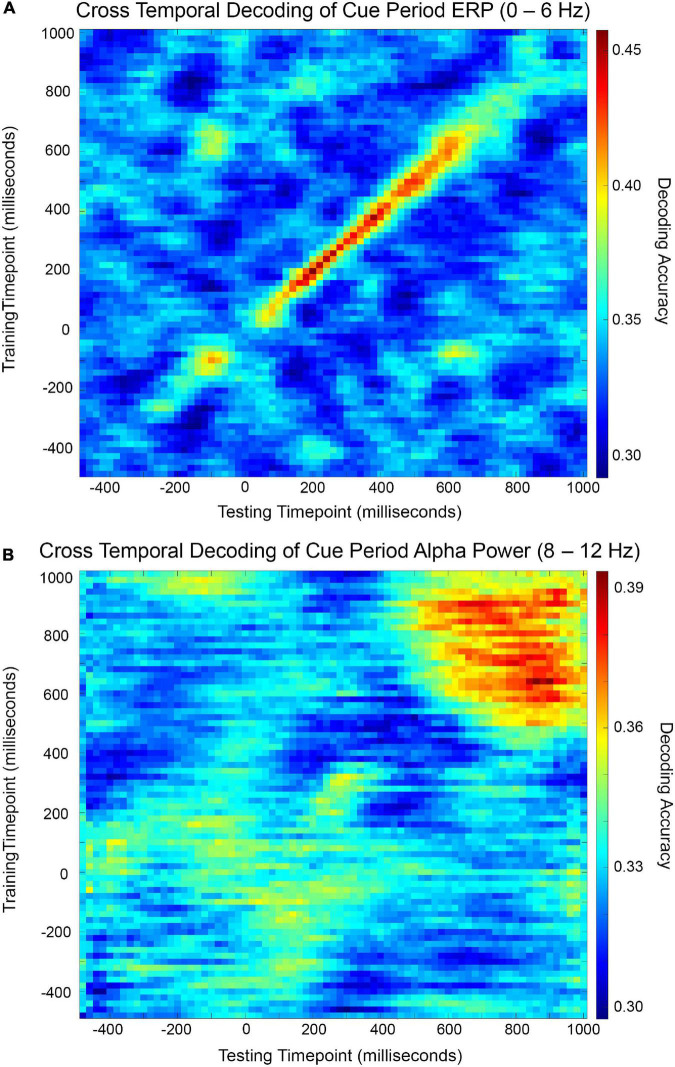
Cross-temporal cue period decoding accuracy matrices for ERP **(A)** and alpha power **(B)**. Decoding accuracy was measured for each combination of training time point and testing time point and visualized by color map.

Cross-temporal decoding matrices are presented in Figure 3. In this decoding approach, a classifier built at time t is used to decode the data from all other time points. As shown in Figure 3A, the cross-temporal ERP decoding matrix reveals that decoding performance off the diagonal fell quickly to chance levels, suggesting transient representation. By contrast, the cross-temporal alpha power decoding matrix (Figure 3B) shows a pattern of relatively long-lasting above-chance off-diagonal decoding performance. Cross-temporal alpha power decoding remained above-chance at comparable levels from the onset of statistically significant decoding, as determined by cluster-based permutation test and visualized in Figure 2B, until the end of the cue period, suggesting stable representation.

## Discussion

The neural mechanisms underlying the control of anticipatory voluntary attention can be subdivided into those belonging to “sources” of biasing signals and “sites” where the top-down biasing influences target processing (Posner and Petersen, 1990; Petersen and Posner, 2012). Top-down attention has been likened to a spotlight (Posner et al., 1980), and the sources of attentional biasing signals are like the lamp, which can be swiveled and pointed toward whatever needs to be selectively illuminated for enhanced visibility. Sensory cortical sites where biasing enhances target representations are like the contents of the spotlight’s beam: their visibility increases because of the lamp’s light.

Sources of top-down attentional biasing signals have been identified in frontal and parietal cortex (Nobre et al., 1997; Corbetta et al., 2000; Hopfinger et al., 2000; Kastner and Ungerleider, 2000). A network comprising intraparietal sulcus, superior parietal lobule, frontal eye field, and supplementary eye field has been identified as an originator of endogenous attentional control signals and labeled as the dorsal attention network (He et al., 2007; Szczepanski et al., 2013). Activity in the dorsal attention network has been linked to modulations of neural activity in sensory areas (Liu et al., 2016; Popov et al., 2017). The role of ventral pre-arcuate region of prefrontal cortex has been identified as a possible source of top-down biasing signals when the target of attention is a naturalistic object (Bichot et al., 2015).

In the visual domain, one potential mechanism by which top-down attention affects processing in sensory cortex is modulation of oscillatory neural activity in the 8–12 Hz alpha band (Scheeringa et al., 2011; Liu et al., 2016). The alpha band may reflect a natural frequency of occipital-corticothalamic circuits (Rosanova et al., 2009; Vallesi et al., 2021). Furthermore, alpha band power within a neural population may reflect the excitability of that population, with lower power indicating higher excitability. Therefore, direct modulation of alpha band power may represent a means by which higher order control regions, such as the dorsal attention network, dynamically regulate the flow of information through various visual cortical pathways to flexibly support behavioral goals (Jensen and Mazaheri, 2010; Van Diepen et al., 2019). Consistent with this theory, systematic alpha power modulations have been observed to accompany attention to spatial locations (Worden et al., 2000), attention to low-level visual features (Snyder and Foxe, 2010), and attention to object categories (Noah et al., 2020), suggesting that alpha power modulation is a common mechanism of attentional control throughout visual cortex.

The hypothesis that alpha synchronization serves as a controllable gate on sensory processing dovetails with findings that anticipatory attention increases firing rates and other correlates of neural activity in sensory cortical areas that are selective for the cued visual information in advance of the actual appearance of the cued stimulus (Luck et al., 1997; Chawla et al., 1999; Kastner et al., 1999; Hopfinger et al., 2000). These findings suggest that anticipatory attention to a target spatial region or visual feature may be subserved by an increase in baseline excitability and spike firing rates in the corresponding cortical processing areas, and that therefore the efficacy of anticipatory attention over a length of time depends on the baseline increase being sustained.

These findings prompt the question of whether sustained increases in sensory cortical baseline activity are dependent on sustained input from attention control regions, or if instead transient inputs from control sources are sufficient to induce steady states of sensory receptivity. The continuous involvement of a network of multiple brain areas in the former scenario would at least be metabolically inefficient (Zenon et al., 2019), and possibly also interfere with the ability of these areas to participate in other computational processes during the anticipatory period (Ophir et al., 2009).

Previous studies of human endogenous attention using fMRI have suggested that attention control signals arising from posterior parietal cortex may be transient, while the effects of sustained attention in visual cortex are sustained (Yantis et al., 2002; Serences and Yantis, 2006). However, the question of whether sustained increases in baseline sensory cortical activity are dependent on sustained activity in the dorsal attention network is not well addressed in fMRI studies (Chawla et al., 1999; Kastner et al., 1999; Hopfinger et al., 2000) because of the low temporal resolution of the hemodynamic signal. In contrast, the millisecond timescale of EEG allows us to address this question with greater temporal precision.

We hypothesize that anticipatory attention does not require continuous maintenance by ongoing input from the dorsal attention network, and is instead a biased, stable state in sensory cortical structures that is induced by transient top-down control signals. Furthermore, we hypothesize that alpha power distributions across visual cortex are endpoints of these top-down control signals, and therefore a cortical alpha power distribution is predicted to be static once it is induced by dynamic control mechanisms.

Based on previous research associating early ERP components with neural processes in frontal and parietal brain areas (Grent-‘T-Jong and Woldorff, 2007), we interpreted our 0–6 Hz ERP band data as encoding attentional control signals. We predicted that control mechanisms would predominantly operate before the onset of systematic modulated alpha power topographies. Thus, we predicted that the ERP signal would be highly dynamic whereas the alpha power topography would be static, and that EEG decoding would reflect this ordering and these characteristics of the ERP and alpha power scalp distributions.

Our SVM decoding results supported our predictions. We observed that in our ERP decoding timeseries, statistically significant above-chance decoding began early in the cue period, peaking around 150–200 ms post-cue, and declining toward chance levels by 750 ms. Statistically significant above-chance decoding of alpha power topographies began near the offset of significant ERP decoding, around 700 ms. That above-chance ERP decoding preceded above-chance alpha power decoding supports our hypothesis that the function of the orienting signals encoded in the ERP is to establish an alpha power topography that supports the selective processing of incoming targeted stimulus information.

We subjected the 0–6 Hz signal to our decoding analysis in the form of instantaneous voltage scalp topographies to capture the cue-related ERP without any overlap from cue-related alpha band responses, following previous EEG decoding analyses that sequestered the 0–6 Hz band for the same purpose (Bae and Luck, 2018). We interpreted the ERP signal as encoding attentional orienting processes. Theoretically, the EEG signal should reflect all the neural activity instigated by cue presentation, including activity underlying visual perception of the cue, interpretation of the cue meaning, and execution of task instructions conveyed by the cue (attentional orienting to object category). The decodability of the cue condition from the EEG signal reflects differences in evoked patterns of brain activity between the different cue conditions, discernible at the scalp. Thus, because different attentional orienting processes are invoked in the different cue conditions, these orienting processes should be reflected in the EEG signal and detectable with EEG decoding.

Our cross-temporal ERP decoding results showed that above-chance decoding did not spread far from the one-to-one training-to-testing diagonal, reflecting that the scalp pattern of ERP voltages encoding the cue-related information changed highly dynamically, near the resolution of our cross-temporal decoding analysis. This dynamism in the cue-related ERP topography suggests that the attentional orienting processes initiated after cue presentation involved transient activity in the neural areas underlying the ERP signal.

We interpret our cross-temporal ERP decoding results to mean that attentional orienting processes in higher order cortical areas compute the appropriate sensory cortical targets to receive modulatory input and issue biasing signals to sensory cortex to enact localized control. Both these computations and the top-down biasing must be encoded in transient neural signals rather than sustained activity, because any sustained activity that varied as a function of our experiment’s cue condition would be reflected in patterns of off-diagonal above-chance accuracy in our cross-temporal ERP decoding matrix. Therefore, our cross-temporal ERP decoding results support our hypothesis that the top-down biasing signal issued from attentional control regions is not a continuous input to sensory cortex.

Our cross-temporal alpha power decoding results indicate that the scalp patterns of alpha power (and therefore the activity of underlying neural generators of these patterns) that varied as a function of cue condition were sustained and highly stable. Unlike our treatment of the 0–6 Hz ERP signal, we modified the 8–12 Hz alpha band signal by Hilbert transform to estimate instantaneous oscillatory power in this frequency band, and thus our alpha decoding reflected alpha power scalp topographies rather than topographies of instantaneous voltages extracted from 8 to 12 Hz activity. That our cross-temporal alpha power decoding remained stably above chance over the roughly 400 ms period from its onset reflects that scalp topographies of power in the alpha frequency band were stable over this period. The brain state associated with this period could therefore be characterized as a resonant system with spatial parameters dictating its functional utility.

Alpha band oscillatory neural activity may reflect an equilibrium state of visual cortical activity. There is evidence that visual cortex settles into an alpha band oscillatory pattern when visual input becomes static (Berger, 1929; Adrian and Matthews, 1934; Pfurtscheller et al., 1996), and furthermore, perturbational studies suggest that the alpha band may be a natural frequency of the visual system (Rosanova et al., 2009; Vallesi et al., 2021). We propose that alpha band activity does reflect a form of cortical inactivity by its nature as a fixed-point behavior. However, unlike historical hypotheses that this inactivity is mere idling and possibly epiphenomenal, we hypothesize that the equilibrium behavior of alpha activity is essential to its functional role. It seems at least plausible that because visual cortical circuits tend toward stable oscillatory behavior in the alpha frequency range (Rosanova et al., 2009; Hindriks et al., 2015; Vallesi et al., 2021), extrinsic inputs to these circuits can modulate the amplitude and phase coherence of synchronized firing by adjusting the system’s initial conditions.

We propose three possible mechanisms by which transient control signals can induce sustained patterns in visual cortex. First, considering that synchronized oscillatory activity in the alpha band may reflect a natural activity pattern of the visual system, top-down control mechanisms may need only act transiently to modulate sustained alpha patterns by either destabilizing alpha band phase coherence in circuits selectively responsive to attended visual features, or conversely, synchronizing phase coherence in circuits selectively responsive to task-irrelevant visual features.

Second, representation of the attentional instruction in visual cortex may be sustained by an activity-silent information maintenance mechanism, by analogy with that proposed to support working memory (Stokes, 2015). Activity-dependent short-term synaptic plasticity is a possible mechanism by which changes in the functional connectivity of a network construct a temporary task-relevant circuit.

Third, higher-order cortical attention signals may be relayed to visual cortex via subcortical control centers. In particular, attentional instruction from frontoparietal control networks may regulate the activity of the pulvinar and in turn facilitate neural communication of attended information in visual cortex by regulating oscillatory local field potential phase coherence in the alpha band (Saalmann et al., 2012). It is unclear whether coordination of communication between visual cortical areas is a result of local thalamic computation or of a distributed network of brain areas, such as an extrathalaic inhibitory system involving the output nuclei of the basal ganglia (Halassa and Kastner, 2017). However, either type of mechanism could theoretically facilitate communication among visual cortical areas after receiving transient frontoparietal control signals.

Altogether, we propose that alpha band activity represents steady visual cortical states and that transient extrinsic inputs can establish different equilibrium behaviors by modulating initial conditions or system parameters. Our proposal bridges historical viewpoints that alpha band oscillations are epiphenomenal signatures of cortical idling with more recent theories about the functional role of this oscillatory regime. This hypothesis might explain why alpha band activity appears to serve a gating function on cortical information processing streams in various cognitive behaviors such as working memory (Bae and Luck, 2018), spatial attention (Worden et al., 2000), feature-based attention (Snyder and Foxe, 2010), and object-based attention (Noah et al., 2020).

A limitation of the data analysis presented here is that it does not support strong claims about causal relationships between the processes reflected by these signals: We observe that ERP signals encoding attentional instructions precede alpha power signals encoding attentional state, but ordering alone is not sufficient to justify a direct causal connection between the two signals. Therefore, future research stemming from this proposal should seek to identify the causal connections between attention control signals and the modulation of alpha band oscillatory activity. For example, future work could seek to model precise mechanisms by which top-down inputs from higher cortical areas and control networks adjust the visual cortical circuit factors that contribute to the establishment of different alpha steady states and identify signatures of these mechanisms in neural data.

Our proposal focuses on alpha band activity to the exclusion of other frequency bands. In previous work, we observed that cued anticipatory object-based attention modulates alpha power topographies, and we did not find any evidence of systematic modulation in similar topographies of theta, beta, or gamma band EEG (Noah et al., 2020). Therefore, here we center alpha band modulation in our theoretical mechanism of anticipatory attention. However, future work testing the hypothesis that modulated alpha power is a steady state phenomenon induced by transient top-down attention control signals should also examine activity in other frequency bands and other electrophysiological signals associated with sustained attention in visual cortex to better understand how modulated alpha power might lead to facilitated cortical computation of task-relevant visual information.

## Conclusion

In this study, we examined ERP and alpha power modulation through the lens of SVM decoding to assess the extent to which these two electrophysiological signals reflect different neural processes underlying the control of object attention. Our results suggested that attentional orienting signals are not continuously applied to maintain a receptive anticipatory state in visual cortex, but rather are dynamic and transient, and the resultant biased visual cortical state was maintained by alpha band oscillations.

We conducted an SVM analysis and cross-temporal decoding analysis to examine the time courses of decoding performance, a proxy measure for cue condition-dependent information in the ERP and alpha power signals, and the stability of the decoded patterns across time. Our results suggest that higher order control signals, encoded in the 0–6 Hz ERP signal, are active before the onset of a stable state reflected by sustained patterns of attention-related alpha power. Furthermore, we propose that to support anticipatory attention, the function of attentional orienting signals originating in higher order sources, such as the dorsal attention network, is to establish conditions in visual cortex so that the system evolves toward an equilibrium state that facilitates the selective reception and transmission of target-related visual information.

The source-site subdivision of endogenous attention’s neural mechanisms has historically likened attention to a spotlight, with one set of neural processes involved in orienting the spotlight and a separate set of mechanisms “illuminating” the representational content of sensory processing areas (Posner et al., 1980). Based on our observation that control mechanisms are transient and the induced attentive state does not rely on continuous energetic input from control sources, we propose that the spotlight metaphor mischaracterizes important aspects of attentional processes. The spotlight metaphor implies that enhancement of sensory representations depends on continuous illumination: once the lamp has been oriented toward the target and the light comes on, it needs to stay on for the target to remain visible.

A new metaphor is suggested by previous lines of fMRI research suggesting the transience of control signals in posterior parietal cortex (Yantis et al., 2002; Serences and Yantis, 2006) and our EEG-based observation of the transience of top-down control signals that instantiate a stable attentional state of visual-cortical alpha power topography. We propose that attention’s implementation in sensory cortex can be thought of more like a radio than a spotlight. The attention control sources are akin to the listener, who tunes the radio to a specific channel. Tuning the radio to the relevant channel facilitates reception of the desired signal, and no other signals on other channels. The act of tuning is a transient event, but the result is sustained.

## Data availability statement

The raw data supporting the conclusions of this article will be made available by the authors, without undue reservation.

## Ethics statement

The studies involving human participants were reviewed and approved by the Institutional Review Board of the University of California, Davis. The patients/participants provided their written informed consent to participate in this study.

## Author contributions

SN conceptualized the study, recorded the data, performed the analyses, and wrote the first draft. SM, MD, and GM contributed to the conceptualization, planning of analyses, and writing of the final manuscript with SN. All authors contributed to the article and approved the submitted version.

## Conflict of interest

The authors declare that the research was conducted in the absence of any commercial or financial relationships that could be construed as a potential conflict of interest.

## Publisher’s note

All claims expressed in this article are solely those of the authors and do not necessarily represent those of their affiliated organizations, or those of the publisher, the editors and the reviewers. Any product that may be evaluated in this article, or claim that may be made by its manufacturer, is not guaranteed or endorsed by the publisher.
